# Precautionary Measures and Contra-Indications to the Use of Pressure by the Tampon in Diseases of the Pelvic Organs

**Published:** 1883-09

**Authors:** V. H. Taliaferro

**Affiliations:** Atlanta, Ga., Professor of Obstetrics and Diseases of Women and Children, in the Atlanta Medical College


ATLANTA
### Medical Register.

Vol. II.] SEPTEMBER, 1883. [No. 12

### Original.

### PRECAUTIONARY MEASURES AND CONTRA-INDICATIONS TO THE USE OF PRESSURE BY THE TAMPON IN DISEASES OF THE PELVIC ORGANS.

By V. H. TALIAFERRO, M.D., Atlanta, Ga.,
Professor of Obstetrics and Diseases of Women and Children, in the Atlanta Medical College.

Some months ago I received a communication from a distinguished medical gentleman, detailing some accidents which had occurred to him in the application of pressure to the pelvic organs by means of the tampon, as first taught by myself in a paper presented to the Medical Association of Georgia, at its annual meeting in April, 1878. So impressed have I been by this letter of inquiry, with the importance of the precautionary measures and the contra-indications attending this method of treatment, that I have felt it my duty, as its author, to caution and guard against accident, and the misuse of an important and powerful factor for good or evil.

From the letter referred to I make the following quotation: "Will you let me ask you have you had mishaps in the shape of cellulitis as the result of your treatment? It must have been two or four years ago that I got your pamphlet in which you recommended packing with wool, and I remember giving it up as unsatisfactory in its results, but on getting your last *brochure*, recommending cotton instead, I concluded the fault must have been in the wool. I think I must have been most careful in the packing on the hands and knees, and in using a pad thoroughly saturated with glycerine and covered with iodoform, as prescribed by you. But I am sorry to say that in one case I produced a most severe and acute attack of cellulitis—in a second and third acute attacks, though not so violent—and in several others such a degree of tenderness approaching inflammation, has resulted, that I have had to intermit the treatment. If I were not so old and experienced a gynecologist, and accustomed to use caution in treatment, I might blame my *modus operandi*. Can you enlighten me? I believe, with you, that *elastic pressure* is THE treatment for chronic indurations and adhesions. Patients always express themselves relieved during the first, second and third packing—then the trouble begins. Can you help me to avoid it?"

In answer to this important, and to me intensely interesting inquiry, I reply:

Your letter of inquiry in reference to the application of pressure by the tampon has been received. I take special interest in replying, inasmuch as your letter has impressed me with the idea that I failed to give sufficient prominence to important and leading facts connected with this method of treatment.

The question as to the *amount of pressure* to be applied to the pelvic organs is one of the first importance, and one always to be considered in every case of pelvic disease in which this method of treatment is applied. We must recognize always the prominent fact that the tampon, if applied to the extent of *decided pressure*, acts primarily as a powerful stimulant to the pelvic vessels, and that consequently inflammatory conditions, both acute and chronic, either *forbid absolutely* the application of pressure by this method, or demaud its use with the *utmost caution*.

Experience has taught me that cases of like pathological conditions, so far as physical exploration can reveal, will not bear with impunity the same degree of pressure. What is comfort and relief for one is discomfort or pain for another. What is safe and even desirable for one is unsafe and even dangerous for another.

Will the tampon cause acute cellulitis? It will. Will the tampon cause pelvic peritonitis? It will. How are these results to be appehended, and how averted? That these results may follow the application of pressure by the tampon does not lessen its value, but indicates its power as a remedy and the necessity of discretion and skill in its application.

In my first paper upon this subject, read before the Medical Association of Georgia in the spring of 1878, it will be found that I had thus early in this method of treatment learned to appreciate the necessity of the utmost caution in its application when there was cause to suspect a lurking inflammation, as will be seen from the following on page 7, eighth line: "It was, at the same time, determined to carefully test the efficacy of *pressure by the tampon* upon the congested and tender uterine organs. In view of the existing blood taint *this must be graduated with the greatest care and delicacy, lest we kindle up an active pelvic inflammation.*" (Italics added).

Again, on page 20, fourth line from top: "Vaginitis, irritable canal, and acute inflammation in the pelvic organs, contra-indicate the tampon, and when they exist should be modified or removed before putting and keeping the canal on the stretch."

Again, on page 20, fifteenth line from bottom: "The vagina should always be, at first, *lightly packed*, or, as I frequently prefer, *partially packed*, filling simply the *vaginal vault.*"

In my paper upon the same subject, published in September, 1882, page 17, commencing thirteenth line from bottom, is found: "If the tenderness of the organs admit it this partial tampon should be *very firm*. If there is considerable tenderness the dressing should be *very light* (italics added), and the pressure gradually increased as the tenderness subsides."

Again, on page 4, third line from bottom: "When the inflammation is *acute* the hot water is preferable and the *tampon contra-indicated.*"

Thus it will be seen that very early in this method of treatment contra-indications were freely recognized and *precautionary measures* specifically pointed out. The letter, however, from which I have quoted, admonishes me that my contra-indications and precautionary measures have not been presented with sufficient force and clearness.

It will be remembered that originally *sheep's wool*, instead of *cotton wool*, was used as a means of pressure to the pelvic organs, otherwise I should, doubtless, have more frequently than has occurred to me, encountered the inflammatory accidents mentioned by the Doctor. Sheep's wool is greatly more elastic and porous than cotton, and cannot, therefore, be packed to make the amount of pressure easily attained by the cotton. It is while using this light and elastic material that I was early led to recognize the importance of *graduating the pressure* to suit individual cases.

Had cotton been the material of my early experiments, instead of the sheep's wool, I should doubtless have more frequently encountered the inflammatory accidents complained of. I was induced to give up the *sheep's wool* for cotton because of its less expense and the greater degree of pressure attainable by it. The cotton packing can be made very firm and solid, and all the pressure made compatible with safety. Not so with the *sheep's wool.* It preserves its elasticity under pressure and moisture, and where light dressing and but little pressure is desired the sheep's wool is best, and were it so cheap and accessible as the cotton, I should use it always where slight pressure and elasticity were desirable.

Now, since we have seen that the application of pressure to the pelvic organs may cause acute pelvic cellulitis, or peritonitis, or both, let us inquire into the special conditions favoring such results. These special conditions will indicate the contra-indications, as also the precautionary measures necessary when pressure is admissible.

Whatever be the method of treatment adopted the first step, the physical exploration of the pelvic organs by the touch, will indicate the knowledge and skill of the gynecologist. The diagnosis must be *absolutely correct*, or the patient may suffer from misapplied practice.

This physiological exploration must determine :

1st. Is there *tenderness* in the periuterine structure?

2d. Is the tenderness dependent upon acute inflammation? Upon chronic inflammation and its products? Upon congestion?

3d. Are there inflammatory deposits and pelvic adhesions?

4th. Is there a history of *recurrent pelvic inflammation?*

5th. Condition of the ovaries.

In these simple questions and their answers we have the key, to the contra-indications and the precautionary measures, to pressure by the tampon.

If the tenderness is dependent upon acute inflammation the tampon is positively contra-indicated. If the tenderness is dependent upon *chronic inflammation*, or its products, *the pressure must be applied with the greatest possible caution.* First, a single pledget with glycerine, and this *gradually and slowly increased*, as the patient will bear it *without pain*, until, in most cases, tolerance is established and pressure may be applied to the extent desired.

In cases with the physical marks, and the history of pelvic inflammation, we should always accompany the tampon with positive instructions *for its removal* in case it causes *continuous pain*, however slight. We should observe the same precaution, here, that we observe in the use of tents to the uterine cavity. Continuous pain calls for the removal of the tent, if we expect to escape inflammation. So, likewise, with the tampon in these cases of latent pelvic inflammation, pain is the signal of danger, and when it occurs the pressure should promptly be removed.

In most cases of inflammatory deposits there will be found a history of *recurrent inflammation.* These recurrent attacks are frequently induced by the slightest causes, and in every case the life of the patient is threatened. These are the causes of all others in which the tampon is *par excellence* the remedy, but its application must be made with the greatest caution, at least until the parts become tolerant of the foreign substance and can bear with impunity the pressure necessary for decided and permanent results. In many cases where *excessive tenderness* is found accompanying these complicated conditions, it may be weeks, or indeed months, before the parts will bear *actual*

*pressure* with impunity. These cases must be *prepared* for *pressure* by disgorging the congested blood vessels with glycerine, as already directed, and the hot-water vaginal douche, alternately. In a little while it will usually be found that the tenderness has so far subsided that the patient will begin to bear a little pressure, which may be gradually increased from time to time, as may be done without pain, until all the pressure desired may be made. The patient should always be instructed that if pain ensues, the dressing must be removed at once and the hot-water vaginal douche substituted. It may be asked of old and extensive pelvic deposits and adhesions, with the history of recurrent acute attacks, if the tampon must be used with so much caution, why use it at all? My reply is *that these conditions can only be remedied by pressure.* Upon these old deposits and adhesions all other remedies within my knowledge are futile. Emmet's hot-water vaginal douche will give comfort, and modify the congestion and tenderness, but nothing more.

In the Doctor's inquiry, whose letter is quoted, it is stated: "In the first, second and third tampon the patient expresses great relief, but after this the trouble comes." In these cases a *lurking inflammation* was overlooked, or the tampon was applied *too firmly in the beginning. In all cases*, whether there be adhesion or not, whether there be tenderness or not, the tampon should be applied lightly at first; *there should be no pressure* until the parts are inured to the foreign substance, and then the pressure should be *gradually applied.*

In cases with a congestive and highly sensitive ovary the same precautionary measures must be observed *in the application of pressure.*

I am quite sure that very many who have used the tampon and abandoned it, have done so because they have applied it too firmly *in the beginning.* The pelvic organs will bear with impunity all the pressure that can be applied to them through the vagina, provided the maximum degree of pressure is attained *gradually.*

In Bozeman's so-called "column" there is no pressure, and hence as little danger of cellulitis as there is of good, and consequently no contra-indication to its use. His method of columnizing the vagina with dry cotton is utterly inefficient, and the results, save in the irritation to the vaginal walls, absolutely *nil.*

It is the *pressure* which makes the tampon a powerful factor in therapeutic results; and the same factor, so potent for good results, is equally potent for injurious sequences when misapplied.

As an evidence of the manner in which my teachings have been followed in very many instances, the following is an example: I was called in consultation some months since to see a lady who for some months had been confined to bed and under the care of an old practitioner and intelligent gentleman. The history he gave me of his case was one of pelvic inflammation, with great suffering. He told me that his patient did not improve under the treatment by my method of tamponade, though persevered in for several months.

I found, by examination, the patient suffering from a retroverted and prolapsed uterus, with extensive deposits and adhesions, the kind of case of all others calling for the tampon. Is it not singular then that the patient has not improved, but actually grown worse? Inquiry developed the fact that the tampon had been applied through a *cylindrical speculum*, with the patient *on the back.* How very singular, since the instructions are so explicit in every detail as to the position of the patient and the *modus operandi* of the tamponade. Yet it is not more singular than other awkward manipulations which have come within my knowledge, by those professing to follow closely this method of treatment.

The difference in nervous organization and local hyper æsthesia will sufficiently explain the difference in effect and results, by the tampon, in patients with like pathological disturbances, so far as physical exploration can determine. In one, the tampon firmly applied gives the greatest possible comfort and enthusiasm in praise of the sudden and decided relief, while in the other the patient complains of constant discomfort, which perhaps increases to constant pain. The fault is not in the tampon, but in our inability to make the nice discriminations in diagnosis necessary to always avoid error.

Patients we thought similar and requiring similar treatment, are dissimilar and require different treatment; one has a hysterical spine or a hysterical ovary, and the other has not; one is *plus* and the other *minus* (if we may so express it) in nervous organization; one has pelvic hyperæsthesia and the other not. It is only by the closest study and observation of individual cases that we are enabled to determine that one patient will bear with safety the introduction of a probe, while in another this simple procedure would jeopardize life; in one a *firm tampon* gives relief, and in another a cellulitis.

Then to answer the Doctor's question as to how to avoid such accidents, I will reply that it can only be done by a thorough comprehension of the scope and power of our remedy, and a full appreciation of the contra-indications to its use. These, together with a thorough education in our diagnostic resources, and the requisite *tactus eruditus* in their application, must be our guides to success and to the avoidance of error.

We must remember, however, that with the *highest grade* of education and skill, that we are still fallible, and accidents and disappointments will befall us. We must not censure the remedy because, forsooth, it sometimes yields us *bad* instead of *good.* We must not abandon ovariotomy because its marked percentage of mortality still exists. So long as its good results vastly outweigh its failures, so long must we rank it a valued curative resource; and so with all remedies and potent methods of treatment.

